# Measurement and modeling of diffuse ultraviolet radiation: A review

**DOI:** 10.1111/php.70084

**Published:** 2026-03-02

**Authors:** Alfio V. Parisi, Joanna Turner, Abdurazaq Amar, Damien P. Igoe, Peter Schouten, Nawin Raj, Nathan Downs, Harry Butler

**Affiliations:** ^1^ School of Science, Engineering and Digital Technologies University of Southern Queensland Toowoomba Queensland Australia; ^2^ UniSQ College University of Southern Queensland Toowoomba Queensland Australia; ^3^ UQ College University of Queensland Brisbane Queensland Australia

**Keywords:** diffuse, erythema, measurement, UV, UVB

## Abstract

The ultraviolet (UV) radiation environment consists of direct UV and diffuse UV components. The ratios of diffuse‐to‐direct UV for the UVB, UVA, and the erythema UV wavebands are influenced by clouds, aerosols, albedo, and surface reflectance, Rayleigh scattering, and solar zenith angle. At times, the relative proportion of diffuse UV may be higher than the direct UV, for example, on cloudy days or days with high atmospheric aerosols. Consequently, exposures due to diffuse UV radiation play a significant role in the UV exposure received by human subjects. Diffuse UV radiation contributes to the risk of skin cancer and sun‐related eye disorders. Reducing personal exposure to diffuse UV by physical protection, including tree shade, purpose‐built shade structures, protection by hats, and eyewear, is more difficult than reducing direct UV exposure due to the diffuse UV being incident from all directions. The UV index reported to the public represents the sum of the direct and the diffuse erythema UV, with no information provided specifically on the diffuse UV component. This paper reviews the factors that contribute to diffuse UV exposures affecting human populations. It examines diffuse UV modeling techniques, and broadband and spectral component measurements.

## Graphical abstract

Solar UV radiation consists of direct and diffuse UV components. The diffuse component contributes to the exposure received by humans and to the risk of skin cancer and sun‐related eye disorders. This review aims to determine future research directions for the measurement and modeling of diffuse solar UV radiation. It reviews the environmental factors that influence the relative proportions of the diffuse and the direct components, along with measurement methods available to measure ambient diffuse UV. The diffuse solar UV affecting humans is presented alongside techniques that have been employed to model the diffuse component in different environments.

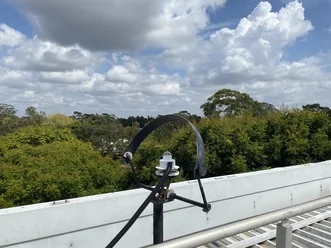

**Abbreviations**
AI: artificial intelligence
FWHM: full width at half maximum
MFRSR: multi‐filter rotating shadow‐band radiometer
PF: protection factor
SED: standard erythemal dose
SZA: solar zenith angle
UV: ultraviolet
UVA: 320–400 nm
UVB: 280–320 nm

## INTRODUCTION

The Rayleigh scattering of ultraviolet (UV) radiation by molecules in the air is inversely proportional to the fourth power of the wavelength. This results in the shorter UVB (280–320 nm) wavelengths being scattered more effectively than the longer UVA (320–400 nm) wavelengths.^1^ Additionally, scattering by clouds and aerosols contributes to the enhancement of diffuse UV radiation,^2^ alongside the scattering due to albedo and reflectance.^3^ These scattering processes result in a significantly higher proportion of diffuse UV radiation in the environment compared with longer wavelength diffuse visible radiation. The damaging biological responses to human skin and eyes, and the synthesis of vitamin D by exposure to sunlight in human skin are significantly higher in the UVB than the UVA waveband,^4^, ^5^, ^6^ making diffuse UV radiation a significant factor contributing to human health.

Depending on specific atmospheric conditions, the UV exposure received by human subjects may be a result of exposure to a greater proportion of diffuse UV rather than direct UV, and in certain scenarios, it can account for up to 80% of the annual UV dose.^7^ Due to the diffuse UV being incident from all directions, it is more difficult to mitigate using conventional protective methods such as hats and shade structures, compared with direct UV. The aim of this narrative review is to determine future research directions for the measurement and modeling of diffuse UV. The objectives for this aim are to review the following areas of research:

- The environmental factors that influence the relative proportions of the diffuse and the direct UV;
- The measurements of the ambient diffuse UV and the diffuse UV affecting populations exposed to solar UV radiation in different environments; and
- The techniques that have been employed to model the diffuse UV.

## METHODS

Google Scholar was used as the primary database for this narrative review. Review topics were developed utilizing the authors' knowledge of existing research. Additional searches employed as required were undertaken in Scopus, Web of Science, ScienceDirect, SpringerLink, Wiley Online Library, JSTOR, and Oxford Academic.

## ENVIRONMENTAL INFLUENCES ON DIFFUSE UV

### Clouds

Cloud and absorbing aerosols are the dominant factors influencing change in the solar UV radiation received by populated regions of the earth.^8^, ^9^ Clouds significantly influence the levels and distribution of solar UV radiation reaching the Earth's surface by modifying both its intensity and distribution through scattering processes. The high spatial and temporal variability of cloud cover presents substantial challenges in accurately quantifying its effects on UV radiation, with changes in cloud structure and position making the radiative influence difficult to model.^2^, ^10^, ^11^ Typically, cloud cover does not significantly influence the UV irradiance until fractions exceed 50% whence the probability of cloud obscuring the sun increases (thus reducing the total irradiance), and scattering effects due to reflections off the side and edges of nearby cloud can modify the radiation received at the surface.^12^

Generally, clouds attenuate direct UV radiation through scattering by suspended water droplets and ice crystals, which reduces atmospheric transmissivity.^2^ Simultaneously, these scattering processes can enhance diffuse UV radiation, depending on factors such as cloud type, optical thickness, obscured sky fraction, and solar position. Consequently, the diffuse fraction of total UV irradiance may increase significantly, particularly when the solar disc is obscured.^13^ The relationship between cloud cover and UV attenuation is non‐linear, as scattering differs between radiation passing through cloud bodies and that reflected off cloud edges.^13^, ^14^

Under full cloud cover, UVB radiation can be reduced by approximately 65–72%, depending on solar elevation.^15^ Overcast skies have also been shown to reduce UVB radiation by up to 80%,^16^ although scattering of short wavelength UVB is typically less than observed scattering by cloud at longer wavelength UVA and total global solar radiation.^17^, ^18^ Observations from a Mediterranean site revealed that the diffuse erythemal UV component ranged from 62% of total UV exposure under clear skies to as much as 93% under heavy cloud conditions.^19^ Similarly, Silva^20^ found that under overcast conditions dominated by cumulus clouds, the diffuse‐to‐global UV irradiance ratio reached 0.99.

Under specific conditions, clouds can amplify surface UV radiation.^2^, ^14^, ^21^ This enhancement occurs when the solar disc remains visible and is surrounded by reflective cloud edges, which increase forward scattering and elevate diffuse UV levels.^2^, ^22^ Maximum cloud‐induced UVB enhancement may result from the combined effect of refraction and scattering of both direct and diffuse sunlight. Specifically, this effect can occur when sunlight is refracted through haze or high‐altitude cirrus clouds across the solar disc, followed by forward scattering from the edges of lower‐altitude clouds such as cumulus.^14^ It has been reported that cloud‐enhanced UVB irradiance occurred in at least 3% of measurements collected at 6‐min intervals over a one‐year period.^14^ Of these enhancement events, 85% occurred when the solar zenith angle ranged between 40° and 63°. The maximum enhancement, 8% above clear‐sky levels, was observed when the solar disc was partially visible and not completely obscured by cloud. Other studies^23^, ^24^, ^25^, ^26^, ^27^ have reported enhancements ranging from 1% to 30%, particularly under cirrus or stratocumulus clouds optimally positioned relative to the sun.

### Aerosols

Aerosols, originating from natural and anthropogenic sources, possess a wide range of absorption and scattering properties. These characteristic effects represent one of the most important factors for accurate UV measurements, particularly under cloudless skies.^28^, ^29^, ^30^, ^31^ Attenuation due to UV absorbing aerosols is a well‐documented important factor that leads to a reduction of UV exposure.^30^, ^32^, ^33^, ^34^, ^35^ Just as important, scattering of the direct UV component by non‐UV absorbing aerosols enhances the diffuse UV component.^31^, ^34^, ^35^, ^36^, ^37^

The variability of sizes of aerosols means that UV radiation can be susceptible to both Rayleigh and Mie scattering.^34^, ^38^ Diffuse UV is often measured as the broadband diffuse fraction of global UV radiation, showing similar diurnal patterns as direct UV, but arriving from all directions in the sky.^39^ Consequently, the diffuse fraction becomes more predominant in high aerosol loads and low solar elevation as Mie scattering at short wavelengths can exceed Rayleigh scattering by gases.^34^, ^39^ The scattering effects of aerosols, however, are highly variable, influenced by solar zenith angle (SZA), meteorological and geomorphological factors including particle size and distribution. As a result, the ratio of diffuse UV to direct UV can vary, with diffuse UV becoming predominant in an aerosol‐laden sky, particularly at low solar elevation.^36^, ^39^ This can potentially increase UV radiation penetration under canopies.^40^

It is important to note that one of the most significant implications of scattering due to aerosols for human health and biological systems is that it results in UV radiation being incident from all directions,^37^, ^38^, ^39^ which has direct implications for sun protection of the skin and eyes. A case study suggests that when smoke haze from bush fires obscured the sun, the diffuse UV exposure was significant enough to potentially contribute to the cumulative erythemal exposure for those performing outside activities during this time.^41^

### Albedo and Surface Reflectance

The contribution of UV albedo and UV surface reflection to diffuse UV radiation profiles in the atmosphere is underexplored and not fully understood. Nevertheless, studies have aimed to quantify changes to exposure linked to albedo.^3^, ^42^, ^43^ These studies have been used in lieu of direct correlation of UV albedo to measured diffuse UV. A consistent definition of UV albedo and/or UV reflectance that accurately portrays the processes involved in the reflection of UV radiation in the atmosphere is challenging to establish, as discussed by Turner and Parisi.^44^

There is a limited amount of recorded and published data on the UV albedo and surface reflectance, and consequently, the correlation between physical UV reflectance and change in atmospheric UV radiation distribution is also limited. It is generally accepted that artificial surfaces tend to reflect more UV radiation than natural surfaces, both spectrally and specularly,^44^ due to differences in particle size within the boundary media.^45^ Exceptions to this pattern include materials such as sand, water, and snow, which have higher reflectivity due to their intrinsic characteristics.^46^, ^47^, ^48^, ^49^, ^50^, ^51^, ^52^, ^53^ Therefore, understanding the composition and characteristics of materials that contribute to UV reflectance is essential. For instance, geographic locations such as coastal, urban, or alpine regions may experience higher relative proportions of diffuse UV radiation compared with other areas, depending on seasonal variations. Distance between the reflective surface and measurement site is not expected to vary UV albedo substantially^44^ except for aerial measurements, where other reflection mechanisms (such as scattering from the boundary layer) tend to overcome the effects of UV albedo and reflectance.^54^, ^55^, ^56^

## DIFFUSE UV MEASUREMENTS

### Broadband diffuse UV measurements

The diffuse UV radiation incident on a horizontal plane is frequently measured using broadband meters. Diffuse UV is measured by shading the sensor from the direct UV using a solar occultation disk or a band.^19^, ^57^ Examples include the Yankee UVB‐1 (Yankee Environmental Systems, MA, USA) radiometer, which employs a disk mounted on a solar tracker to shade the sensor from direct UV.^19^ Alternatively, a semicircular band is used to shade the expected solar path, with periodic adjustments throughout the year to maintain shading.^58^ A correction may be applied to account for the portion of diffuse UV that is shaded by the band.^59^, ^60^ A comparison of one‐minute diffuse UVB data collected using both disk and band methods has shown good agreement between both measurement systems.^57^

Other studies have mounted the sensor on a solar tracker, with the sensor shaded by a ball.^60^, ^61^, ^62^ Another approach involves measuring direct UV and the global UV (direct plus diffuse UV) and then calculating the diffuse UV as the difference of these quantities. In these applications, direct UV has been measured by employing a small field of view collimator mounted on a solar tracker.^19^

### Diffuse UV measurements at specific wavelengths

The diffuse UV at specific wavelengths can be measured using instruments that capture the UV spectrum, or radiometers equipped with narrow pass band filters. Examples of spectroradiometers include the Bentham spectroradiometer^63^, ^64^ and the Brewer spectrophotometer.^65^ The Bentham spectroradiometer can be configured to measure the direct UV spectrum using a collimator and solar tracker, alongside measurements of the global UV spectrum, enabling the diffuse UV component to be calculated.^34^ The Bentham spectroradiometer can also be equipped with a shadow band that swings into position to shade the input diffuser, enabling direct measurement of the diffuse UV spectrum at 0.5 nm intervals.^66^ Spectral diffuse UV data have also been collected using a Brewer spectrophotometer fitted with a shadow ball mounted on a frame to block the direct UV.^67^

Another approach involves measuring diffuse UV at specific wavelengths using narrow bandwidth filters. For instance, the Biospherical Instruments radiometer (model GUV‐511 C) has been employed to measure both the global irradiance at 305, 320, 340, and 380 nm with a bandwidth of 10 nm, and the diffuse UV at these wavelengths with a software controlled shadow band that swings into position to shade the input sensor.^68^ Similarly, the Multi‐Filter Rotating Shadow‐band Radiometer (MFRSR) undertakes measurements at 300, 305, 311, 317, 325, 332, and 368 nm, with a full width at half maximum (FWHM) of 2 nm.^69^, ^70^ Diffuse UV measurements with this instrument are obtained by using a shadow band that swings into position to periodically obscure the solar disc.

### Diffuse UV measurements in different settings

UV exposures received in shaded environments, such as exposure received under tree shade, umbrellas, and shade structures are predominantly due to the diffuse UV component. Underwater, UV propagation is further affected by attenuation and scattering processes. The measurement of diffuse UV in these different settings plays an important role in contributing to lifetime exposures received by human subjects interacting within the urban environment and during recreational activities.

#### Diffuse UV measurements in tree shade

Techniques employing models and the direct measurement of diffuse solar UV have been applied to evaluate the protection offered by tree shade. One approach involved using a portable data logging meter worn on the shoulder to measure diffuse UV in the shade of five tree species in Durham (United Kingdom).^71^ An electronic logging device (SunSense, Norway) measuring the erythemal UV has been deployed on a horizontal plane in tree shade in California.^72^ In Toronto, Canada, electronic dosimeters (Scienterra, Otago, New Zealand) logging the erythemal UV every minute have been employed to record exposures between 11 am and 2 pm under 64 trees of 16 different species in schools and parks.^73^

Hand‐held or portable radiometers (Solar Light Co., PA, USA) are often used to measure erythemal UV in tree shade. Examples include measurements made in south‐east Queensland,^74^, ^75^, ^76^ as well as spectral measurements with a dual grating monochromator spectroradiometer (model DH10, Jobin Yvon, France).^77^ Passive dosimeters have also been employed, including spore film dosimeters (VioSpor Blue Line Type II Dosimeter; Bio‐Sense, Bornheim, Germany) weighted to the erythemal UV on a horizontal plane under two trees between 9.30 am and 3.30 pm at Paterna (Valencia, Spain).^78^ Polysulphone dosimeters, calibrated to record cumulative erythemal UV exposure, have been used in Adelaide, Australia, under six tree species for 2 h centered around solar noon at a height of 0.5 m.^79^

To assess erythemal exposures to different human anatomical sites, polysulphone dosimeters have been mounted on an upright manikin placed on a rotating platform to simulate a standing human in the shade of Australian gum trees (*Eucalyptus* sp.) in Toowoomba, Australia.^80^, ^81^

#### Diffuse UV measurements in artificial shade

Similar to the measurement of the diffuse UV in tree shade, various techniques have been employed to measure diffuse UV under artificial built shade structures. Diffuse erythemal UV in the shade of sun‐umbrellas has been measured on a horizontal plane using electronic sensors (SunSense, Norway) to record erythemal UV exposures and irradiances,^72^ as well as with a spectroradiometer (model SR9910‐PC, Macam Photometrics, Livingstone, Scotland) and portable radiometers (model 501A, Solar Light Co., PA, USA).^82^ Erythemal UV and the UVA have been measured on a horizontal plane 0.41 meters above ground level using a hand‐held UV meter (model 3D, Solar Light Co., PA, USA) under three differently sized shade structures in public parks in Toowoomba, Australia.^83^, ^84^ Another UV meter fitted with UVB and UVA detectors (model IL1400A, International Light, MA, USA) has been used to measure the UV in various shade settings in Townsville, Australia.^85^

In Lubbock, Texas, 10 electronic dosimeters (Scienterra, Otago, New Zealand) were worn on the wrist of school children for 6 days during after school physical activity to measure erythemal UV exposure in both open areas and in the shade of a large sail structure.^86^ Another approach involved using polysulphone dosimeters to measure erythemal UV under shade structures at10 schools 1 m above the ground on a horizontal and vertical plane across six cities in New Zealand.^87^ A range of studies have been designed to measure the UV irradiance under artificial shade in comparison with environments not protected by shade. Dobbinson et al.^88^ measured reductions averaging almost 3 SED during periods of high solar elevation in metropolitan parkland environments protected by shade sails compared with similar recreational areas not protected by shade. Turnbull and Parisi^89^ showed that exposure to diffuse UV under shade structures can have benefits for vitamin D production. Similar derivations of the ultraviolet protection factor (PF) have been made by comparing measurements of the diffuse UV radiation under shade to the ambient global UV available in an open area (for example^83^, ^90^, ^91^).

#### Underwater UV scattering

UV is relevant as it contributes to the UV exposures of aquatic organisms and human population groups including recreational swimmers and snorkellers. Like terrestrial solar UV radiation, UV radiation propagating within natural bodies of water is influenced by both absorption and scattering processes. The extent to which these processes affect underwater UV irradiance depends on the type and concentration of materials suspended in the water. The propagation of radiation in water, accounting for absorption and scattering, can be modeled by an exponential Equation^92^:

$$E\left(z,\lambda \right)=E\left(0,\lambda \right)e^{-K\left(z,\;\lambda \right)z},$$

where *z* is the depth below the water surface, *K(z,λ)* is a total attenuation coefficient, *E(0,λ)* is the spectral irradiance measured at a depth just below the water line, and *E(z,λ)* is the spectral irradiance at depth *z*. Irradiance can be measured as either a downwelling component *E*_*d*_ or an upwelling component *E*_*u*_. Consequently, the equation used to evaluate *K(z,λ)* can be rewritten to evaluate either the downwelling *K*_*d*_ or upwelling *K*_*u*_ attenuation coefficients. Most research on underwater UV radiation focuses on measuring values for *K*_*d*_.

Generally, clear waters with minimal levels of turbidity have smaller characteristic *K*_*d*_ compared with turbid waters, which are typically inhabited by various forms of animal and plant life that contribute to both absorption and scattering processes. *K*_*d*_ approximations in turbid waters can be influenced by the presence of particulate and dissolved materials, such as dissolved organic matter (DOM) and phytoplankton.^93^ The exponential formula describing the propagation and attenuation of irradiance with depth in water can be expressed in logarithmic form, and *K*_*d*_ can be found by plotting the natural logarithm of the irradiance ratio against depth and calculating the absolute gradient of the resultant line of best fit. This method has been used to estimate *K*_*d*_ values in a range of different water types using UV‐sensitive dosimeters and radiometers.^94^, ^95^

Accurate measurements of underwater solar UV irradiance and *K*_*d*_ estimations are challenging due to several factors: unwieldy instrumentation, limited spectroradiometer/radiometer sensitivity, unpredictable and rapid changes in water quality from natural and anthropogenic activity, shading from aquatic vegetation, random surface wave action, wave focusing effects, and fluctuations in waterline elevation caused by tides and evaporation.^96^, ^97^ To address these challenges, several investigations^95^, ^98^, ^99^ have developed novel in‐situ and laboratory‐based measurement techniques. Underwater measurements that take into account contributions from direct and diffuse UV components need to be corrected for the immersion effect, which arises from the difference between the refractive indices between water and air. In water, a higher proportion of radiation is scattered away from the detector compared with air, resulting in reduced incident radiation and lower measured values.^100^ Numerous studies have developed procedures on how to correct underwater radiation measurements for the immersion effect.^101^, ^102^, ^103^

## MODELING OF DIFFUSE UV

### Radiative transfer, semi‐empirical, and empirical models

Modeling of the UV is important in order to fill spatial and temporal gaps in measurements as well as enabling measurements of the surface irradiance to be checked against model outputs. Direct and diffuse UV components incident on a horizontal plane can be calculated employing semi‐empirical models.^104^, ^105^, ^106^ These models account for variables such as solar zenith angle (SZA), atmospheric ozone, aerosols, and the assumed albedo of ground and vertical surfaces. Empirical models and semi‐empirical models based on cloud cover measurements^13^ as well as aerosols and SZA have been developed to evaluate the diffuse UV under partly cloudy conditions.^61^ Radiative transfer models have also been developed and employed for diffuse UV calculations.^107^, ^108^ One widely used example is the radiative transfer simulation tool UVSPEC, which is part of the libRadtran (library for radiative transfer) package. UVSPEC has been extensively employed to simulate UV irradiance under various atmospheric conditions.^107^, ^109^ Another approach involves segmenting measured erythemal UV into its direct and diffuse components. This was achieved using a linear model with constant cloud optical depth and a non‐linear empirical model relating diffuse erythemal UV to cloud conditions.^59^ Sky view models have also been employed to estimate UV exposure under shading elements such as umbrellas,^110^ suburban tree canopies,^111^, ^112^ and shading installations.^113^

Some of these models have been integrated with models of the human form, segmented into small elements to calculate irradiance and exposure at different anatomical sites.^114^ One such model, SimUVEx, has been developed and tested to incorporate direct, diffuse, and ground‐reflected UV components to evaluate the UV exposure across five body postures.^115^ Another approach for translating modeled direct and diffuse UV on a horizontal plane to specific sites on an upright human involves the use of exposure ratios. These ratios, determined through dosimetric measurements, correspond to SZA ranges of 0°–30°, 30°–50°, and 50°–80°, allowing their use at different latitudes and seasons.^116^ Models of the diffuse UV have been applied to simulate UV irradiances in a variety of environments including the calculation of diffuse UV under changing cloud cover conditions^108^ and aerosol loads.^34^ Calculation of the surface UV irradiance under tree shade and purpose‐built shade structures needs to take into account the contribution of diffuse UV radiation. Typically, this involves an evaluation of the unobstructed sky view visible at a site of interest and the derivation of the diffuse contribution to the total UV irradiance at different SZA and sky conditions. Examples include modeling diffuse UV exposures in urban streetscapes,^112^, ^117^ forests,^118^ school settings,^119^, ^120^ public parks,^121^ and under shade structures.^89^, ^113^

### AI models

Diffuse UV radiation is influenced by many atmospheric variables,^122^ making accurate and reliable predictions particularly challenging. Many of these variables interact in a highly non‐linear pattern, which can limit the effectiveness of conventional mathematical models in representing the full range of variables.^123^, ^124^ Furthermore, UV radiation and atmospheric variables are highly sensitive to initial conditions, meaning small errors in initial state could lead to high errors in predictions.^125^ In addition, the high temporal and spatial variability further contributes to the complexity of the problem.^126^

Hence, as an alternative to the conventional mathematical approaches, data‐driven methods have been developed and adopted in recent times. The advancement of AI modeling platforms and their ability to process large datasets in relatively short time periods has significantly improved the accuracy of diffuse UV forecasting.^127^ This technological advantage, combined with high computational efficiency, has allowed for the input of many atmospheric factors into the AI model for the extraction of important non‐linear relationships for training.^128^ Various studies have employed AI models for short‐ and long‐term diffuse UV forecasting, including standalone architecture such as artificial neural network (ANN)^66^, ^129^, ^130^ as well as more advanced hybrid deep learning models such as convolutional neural network‐long short‐term memory (CNN‐LSTM).^131^ These studies have focused on various inputs which play a role as feature inputs with diffuse UV as the target for both short‐ and long‐term forecasting.

AI models have been extended to predict the 10‐, 30‐, and 60‐min UV index^131^ and have the potential to forecast the diffuse UV over the same time frames. A data‐driven extreme learning machine algorithm was developed to forecast the UV index utilizing global and diffuse UV at changing SZA.^66^ A short‐term solar UV radiation forecasting Bayesian optimized interpretable artificial intelligence model has also been developed,^132^ achieving high accuracy in predicting cloud‐affected UVA. Another study developed 36 models using three machine learning models to forecast diffuse irradiance to overcome site‐specific and time‐bound limitations for 10‐, 60‐min, and daily timescales.^133^ A study in Nigeria^134^ utilized 12 years meteorological data to compare the hourly diffuse solar radiation predictions using three machine learning models, artificial neural network (ANN), convolutional neural network (CNN), and recurrent neural network (RNN). The study used hourly timesteps using a cloud‐free dataset and found RNN to be most effective with a prediction accuracy of 0.95.

To improve the accuracy of UV irradiance forecasting (including the diffuse UV component) while avoiding overfitting, numerous studies have employed hybrid methodologies that combine radiative transfer models with advanced machine learning techniques. One such study^135^ integrated a radiative transfer model and machine learning techniques including random forest (RF), extreme gradient boosting (XGBoost), multi‐layer perceptron (MLP), deep neural networks (DNN), and convolutional neural networks (CNN). A similar approach which used average daily solar irradiance data derived from their radiative transfer model (RTM + MODIS + meteorological inputs) and integrated with Residual Networks (ResNet) was applied over the Arabian Peninsula,^136^ attaining an R^2^ value of 0.99 for the daily timescale. A physics‐guided neural network model using Himawari‐8 top‐of‐atmosphere (TOA) reflectance and angular data was also studied for hourly and daily diffuse solar radiation estimates,^137^ combining the advantages of the radiative transfer model and deep learning to estimate diffuse global solar radiation.

## CONCLUSION

The diffuse UV comprises a high proportion of the population's overall UV exposure, contributing to the risk of skin cancer, sun‐related eye disorders, and vitamin D synthesis. In shaded environments, UV exposures are primarily due to the diffuse component, which arises from atmospheric scattering as well as reflection and scattering by ground surfaces in the built and natural environment. Despite existing knowledge on UV albedo and reflectance, the absolute contribution of UV albedo or UV reflectance to the total diffuse UV remains poorly quantified. The influence of clouds on the diffuse UV is also complex, owing to the high spatial and temporal variability of cloud cover. Aerosol‐laden skies also affect the relative proportion of the diffuse UV component, with the scattering influenced by solar zenith angle, meteorological conditions, and geomorphological factors.

This review has examined the broadband and spectral measurements of diffuse UV in tree shade, artificial shade, the built, and underwater environments. It has also considered factors involved in modeling diffuse UV for a range of scenarios. A search on Google Scholar of the number of papers found with the search terms “diffuse solar UV” and “Ultraviolet Index” returned approximately 10.8% of the papers for the first term compared with the second term. A refinement of the Google Scholar search for “diffuse solar UV” to “diffuse solar UV skin cancer prevention” returned approximately 4.6% of the “diffuse solar UV” papers. Given the complexity of the processes influencing diffuse UV in various settings where the general public may be exposed, further research is evidently needed on the measurement and modeling of biologically weighted diffuse UV, diffuse UVB, and diffuse UVA. Research is also required to explore the feasibility of providing the public with forecasts of diffuse UV and real‐time diffuse UV irradiance data.

### Recommendations

- There is an evident need for improved ground‐based diffuse solar UV radiation measurement and monitoring. Global changes in UVB and erythema weighted solar UV are the result of changes to global cloud and aerosol distribution. Under an increased and changing cloud cover and aerosol climate scenario, diffuse solar UV will develop as an increasingly dominant component of the potentially harmful solar radiation affecting at‐risk populations.
- Discretized measurement of the direct and diffuse solar UV will play a valuable role in determining further changes to solar radiation affecting the global environment and local populations.
- Protection from diffuse solar UV radiation should be highlighted by prevention strategies aimed at reducing skin cancer risk and global eye disease. Awareness of the harm caused by radiation emanating from the surrounding sky has the potential to inform future sun awareness campaigns. An awareness of the potential for diffuse radiation to cause harm has not previously been highlighted in the literature. This is clearly an avenue for future research.

## Data Availability

The data that support the findings of this study are available in Google Scholar.
